# Supplementary material for: Multi‐dataset identification of innovative feature genes and molecular mechanisms in keratoconus
Source: J Cell Mol Med. 2024 Sep 19;28(18):e70079. doi: 10.1111/jcmm.70079 (PMC11412914; doi:10.1111/jcmm.70079)
Supplement: Supplementary file 2 — Material S2. [file JCMM-28-e70079-s002.zip › 7.GSEA analysis/1.ARL11/KEGG_NEUROTROPHIN_SIGNALING_PATHWAY.html]

Details for gene set KEGG\_NEUROTROPHIN\_SIGNALING\_PATHWAY[GSEA]

|  || Dataset | ARL11.group.cls#h\_versus\_l |
| Phenotype | group.cls#h\_versus\_l |
| Upregulated in class | l |
| GeneSet | KEGG\_NEUROTROPHIN\_SIGNALING\_PATHWAY |
| Enrichment Score (ES) | -0.20111924 |
| Normalized Enrichment Score (NES) | -0.99868166 |
| Nominal p-value | 0.43678162 |
| FDR q-value | 1.0 |
| FWER p-Value | 1.0 |
Table: GSEA Results Summary

  

Fig 1: Enrichment plot: KEGG\_NEUROTROPHIN\_SIGNALING\_PATHWAY      
 Profile of the Running ES Score & Positions of GeneSet Members on the Rank Ordered List

  

| SYMBOL | TITLE | RANK IN GENE LIST | RANK METRIC SCORE | RUNNING ES | CORE ENRICHMENT || 1 | PIK3R3 | na | 2180 | 0.284 | -0.0331 | No |
| 2 | PIK3CG | na | 2870 | 0.247 | -0.0271 | No |
| 3 | CALML6 | na | 3001 | 0.240 | -0.0056 | No |
| 4 | NGFR | na | 3348 | 0.225 | 0.0081 | No |
| 5 | CAMK2B | na | 3930 | 0.203 | 0.0127 | No |
| 6 | PLCG1 | na | 4334 | 0.189 | 0.0209 | No |
| 7 | SHC3 | na | 4454 | 0.184 | 0.0368 | No |
| 8 | NTRK1 | na | 4617 | 0.179 | 0.0509 | No |
| 9 | PIK3R1 | na | 6079 | 0.135 | 0.0230 | No |
| 10 | PLCG2 | na | 6096 | 0.135 | 0.0367 | No |
| 11 | SOS1 | na | 6194 | 0.132 | 0.0478 | No |
| 12 | CAMK2A | na | 6879 | 0.114 | 0.0401 | No |
| 13 | RPS6KA5 | na | 7067 | 0.110 | 0.0463 | No |
| 14 | IKBKB | na | 8698 | 0.079 | 0.0075 | No |
| 15 | PIK3R2 | na | 8783 | 0.078 | 0.0133 | No |
| 16 | RPS6KA2 | na | 8887 | 0.077 | 0.0184 | No |
| 17 | AKT2 | na | 9348 | 0.070 | 0.0124 | No |
| 18 | TP53 | na | 9447 | 0.068 | 0.0168 | No |
| 19 | SOS2 | na | 9955 | 0.061 | 0.0085 | No |
| 20 | PIK3R5 | na | 10118 | 0.059 | 0.0100 | No |
| 21 | MAP3K3 | na | 10136 | 0.059 | 0.0157 | No |
| 22 | KIDINS220 | na | 10140 | 0.059 | 0.0218 | No |
| 23 | BRAF | na | 10628 | 0.052 | 0.0132 | No |
| 24 | KRAS | na | 10630 | 0.052 | 0.0187 | No |
| 25 | PDPK1 | na | 10673 | 0.052 | 0.0229 | No |
| 26 | MAPK9 | na | 11167 | 0.046 | 0.0134 | No |
| 27 | CAMK2G | na | 11259 | 0.045 | 0.0155 | No |
| 28 | MAPK11 | na | 11276 | 0.044 | 0.0197 | No |
| 29 | MAPK7 | na | 11510 | 0.041 | 0.0173 | No |
| 30 | SORT1 | na | 11564 | 0.041 | 0.0201 | No |
| 31 | YWHAE | na | 11573 | 0.041 | 0.0241 | No |
| 32 | CSK | na | 11826 | 0.038 | 0.0208 | No |
| 33 | IRAK1 | na | 12272 | 0.034 | 0.0115 | No |
| 34 | MAP3K1 | na | 12394 | 0.032 | 0.0114 | No |
| 35 | RPS6KA4 | na | 12471 | 0.032 | 0.0126 | No |
| 36 | YWHAB | na | 12974 | 0.027 | 0.0009 | No |
| 37 | TP73 | na | 13053 | 0.027 | 0.0015 | No |
| 38 | MAPK12 | na | 13078 | 0.026 | 0.0035 | No |
| 39 | JUN | na | 13319 | 0.024 | -0.0008 | No |
| 40 | PIK3CA | na | 13512 | 0.023 | -0.0040 | No |
| 41 | MAP2K5 | na | 14281 | 0.017 | -0.0244 | No |
| 42 | FRS2 | na | 14442 | 0.016 | -0.0274 | No |
| 43 | YWHAZ | na | 14484 | 0.015 | -0.0270 | No |
| 44 | CALM2 | na | 14606 | 0.014 | -0.0290 | No |
| 45 | PTPN11 | na | 14607 | 0.014 | -0.0275 | No |
| 46 | MAP2K7 | na | 14659 | 0.014 | -0.0275 | No |
| 47 | ATF4 | na | 14783 | 0.013 | -0.0297 | No |
| 48 | MAPK10 | na | 15299 | 0.009 | -0.0437 | No |
| 49 | IRAK3 | na | 15519 | 0.007 | -0.0492 | No |
| 50 | FASLG | na | 15958 | 0.004 | -0.0615 | No |
| 51 | RAC1 | na | 16390 | 0.001 | -0.0738 | No |
| 52 | ARHGDIB | na | 16494 | 0.000 | -0.0767 | No |
| 53 | NTF4 | na | 16572 | -0.000 | -0.0789 | No |
| 54 | RPS6KA3 | na | 16678 | -0.001 | -0.0819 | No |
| 55 | PRKCD | na | 16865 | -0.002 | -0.0870 | No |
| 56 | PSEN1 | na | 16871 | -0.002 | -0.0869 | No |
| 57 | PRDM4 | na | 16895 | -0.002 | -0.0873 | No |
| 58 | MAP2K2 | na | 16997 | -0.003 | -0.0899 | No |
| 59 | CALML5 | na | 17116 | -0.004 | -0.0929 | No |
| 60 | MAPK14 | na | 17567 | -0.007 | -0.1051 | No |
| 61 | BAX | na | 17601 | -0.008 | -0.1053 | No |
| 62 | RAP1B | na | 17707 | -0.008 | -0.1074 | No |
| 63 | AKT1 | na | 18085 | -0.011 | -0.1172 | No |
| 64 | CDC42 | na | 18361 | -0.013 | -0.1238 | No |
| 65 | HRAS | na | 18435 | -0.013 | -0.1245 | No |
| 66 | CALML3 | na | 18646 | -0.015 | -0.1290 | No |
| 67 | FOXO3 | na | 18679 | -0.015 | -0.1284 | No |
| 68 | CALM1 | na | 18762 | -0.015 | -0.1291 | No |
| 69 | RAP1A | na | 18999 | -0.017 | -0.1342 | No |
| 70 | BAD | na | 19342 | -0.019 | -0.1420 | No |
| 71 | SH2B2 | na | 19646 | -0.021 | -0.1485 | No |
| 72 | YWHAG | na | 19666 | -0.022 | -0.1468 | No |
| 73 | ABL1 | na | 19735 | -0.022 | -0.1465 | No |
| 74 | GSK3B | na | 20728 | -0.029 | -0.1721 | No |
| 75 | MAPK13 | na | 20970 | -0.031 | -0.1758 | No |
| 76 | GAB1 | na | 20972 | -0.031 | -0.1725 | No |
| 77 | TRAF6 | na | 21028 | -0.032 | -0.1708 | No |
| 78 | SHC1 | na | 21475 | -0.035 | -0.1800 | No |
| 79 | ZNF274 | na | 21823 | -0.038 | -0.1860 | No |
| 80 | RHOA | na | 21886 | -0.038 | -0.1838 | No |
| 81 | RPS6KA1 | na | 22083 | -0.041 | -0.1852 | No |
| 82 | IRS4 | na | 22518 | -0.045 | -0.1930 | No |
| 83 | MAPK3 | na | 22614 | -0.046 | -0.1908 | No |
| 84 | CALM3 | na | 22971 | -0.050 | -0.1959 | Yes |
| 85 | YWHAQ | na | 23039 | -0.050 | -0.1925 | Yes |
| 86 | GRB2 | na | 23043 | -0.051 | -0.1873 | Yes |
| 87 | IRAK4 | na | 23085 | -0.051 | -0.1831 | Yes |
| 88 | RAF1 | na | 23336 | -0.054 | -0.1847 | Yes |
| 89 | SH2B1 | na | 23468 | -0.055 | -0.1826 | Yes |
| 90 | MAGED1 | na | 23760 | -0.059 | -0.1848 | Yes |
| 91 | IRAK2 | na | 23774 | -0.060 | -0.1789 | Yes |
| 92 | NFKBIB | na | 23780 | -0.060 | -0.1728 | Yes |
| 93 | PIK3CB | na | 23818 | -0.060 | -0.1675 | Yes |
| 94 | CAMK4 | na | 23937 | -0.061 | -0.1645 | Yes |
| 95 | RPS6KA6 | na | 24013 | -0.062 | -0.1601 | Yes |
| 96 | BCL2 | na | 24069 | -0.063 | -0.1550 | Yes |
| 97 | NFKBIA | na | 24454 | -0.069 | -0.1589 | Yes |
| 98 | ARHGDIA | na | 24480 | -0.069 | -0.1523 | Yes |
| 99 | MAPK8 | na | 24525 | -0.070 | -0.1463 | Yes |
| 100 | MAPK1 | na | 24626 | -0.071 | -0.1417 | Yes |
| 101 | NTRK2 | na | 25301 | -0.080 | -0.1527 | Yes |
| 102 | CRK | na | 25703 | -0.086 | -0.1553 | Yes |
| 103 | PIK3CD | na | 25759 | -0.087 | -0.1478 | Yes |
| 104 | MAPKAPK2 | na | 25894 | -0.089 | -0.1424 | Yes |
| 105 | RAPGEF1 | na | 26203 | -0.093 | -0.1414 | Yes |
| 106 | NTF3 | na | 26592 | -0.100 | -0.1421 | Yes |
| 107 | MAP3K5 | na | 26641 | -0.101 | -0.1328 | Yes |
| 108 | NFKB1 | na | 26767 | -0.104 | -0.1255 | Yes |
| 109 | NTRK3 | na | 27294 | -0.115 | -0.1286 | Yes |
| 110 | AKT3 | na | 27547 | -0.120 | -0.1233 | Yes |
| 111 | NRAS | na | 27790 | -0.126 | -0.1171 | Yes |
| 112 | NFKBIE | na | 27794 | -0.126 | -0.1040 | Yes |
| 113 | CRKL | na | 27878 | -0.128 | -0.0929 | Yes |
| 114 | RIPK2 | na | 28368 | -0.140 | -0.0923 | Yes |
| 115 | YWHAH | na | 28495 | -0.143 | -0.0809 | Yes |
| 116 | RELA | na | 29186 | -0.162 | -0.0838 | Yes |
| 117 | SHC2 | na | 29625 | -0.176 | -0.0779 | Yes |
| 118 | IRS2 | na | 29852 | -0.183 | -0.0652 | Yes |
| 119 | BDNF | na | 30179 | -0.194 | -0.0542 | Yes |
| 120 | MAP2K1 | na | 31070 | -0.223 | -0.0565 | Yes |
| 121 | NGF | na | 31452 | -0.237 | -0.0425 | Yes |
| 122 | IRS1 | na | 32036 | -0.261 | -0.0319 | Yes |
| 123 | CAMK2D | na | 32170 | -0.268 | -0.0075 | Yes |
| 124 | SH2B3 | na | 32257 | -0.272 | 0.0186 | Yes |
| 125 | SHC4 | na | 34410 | -0.501 | 0.0092 | Yes |
Table: GSEA details [plain text format]

  

Fig 2: KEGG\_NEUROTROPHIN\_SIGNALING\_PATHWAY      
 Blue-Pink O' Gram in the Space of the Analyzed GeneSet

  

Fig 3: KEGG\_NEUROTROPHIN\_SIGNALING\_PATHWAY: Random ES distribution      
 Gene set null distribution of ES for **KEGG\_NEUROTROPHIN\_SIGNALING\_PATHWAY**
